# Validation of 2D Force Measurement Roller Ski and Practical Application

**DOI:** 10.3390/s22249856

**Published:** 2022-12-15

**Authors:** Shuang Zhao, Vesa Linnamo, Keijo Ruotsalainen, Stefan Lindinger, Timo Kananen, Petri Koponen, Olli Ohtonen

**Affiliations:** 1 Faculty of Sport and Health Sciences, University of Jyväskylä, 40014 Jyväskylä, Finland; 2 Center of Health and Performance (CHP), Department of Food and Nutrition and Sport Science, University of Gothenburg, 40530 Gothenburg, Sweden; 3 Technical Research Centre of Finland, VTT MIKES, 87100 Kajaani, Finland

**Keywords:** cross-country skiing, force measuring device, kinetics

## Abstract

Several methods could be used to measure the forces from skis or roller skis in cross-country skiing. Equipment that could measure medio-lateral forces may be of good help for investigating the relevant skating techniques. The aim of this study was to validate a pair of newly designed two-dimensional force measurement roller skis. The vertical and medio-lateral forces which were perpendicular to the body of the roller ski could be measured. Forces were resolved into the global coordinate system and compared with the force components measured by a force plate. A static and dynamic loading situation for the force measurement roller ski was performed to reveal the validity of the system. To demonstrate whether the force measurement roller ski would affect roller skiing performance on a treadmill, a maximum speed test with the V2 technique was performed by using both normal and force measurement roller skis. The force-time curves obtained by these two different force measurement systems were shown to have high similarity (coefficient of multiple correlations > 0.940). The absolute difference for the forces in the X and Z directions over one push-off cycle was 3.9–33.3 N. The extra weight (333 g) of the force measurement roller ski did not affect the performance of the skiers. Overall, the newly designed two-dimensional force measurement roller ski in this study is valid for use in future research during daily training for skate skiing techniques.

## 1. Introduction

Numerous tools are available to researchers for the measurement of ground reaction forces (GRFs). In cross-country (XC) skiing, one approach to measuring the GRFs between skis and snow in early studies was by using the force measurement systems buried under snow [1,2,3,4]. These systems allow skiers to ski freely on snow while recording the force data. However, only two or three ski contacts could be measured for one trial with classic-style XC skiing due to the length and construction of the force plate [1,2,3]. The system introduced by Leppävuori [4] could be used to measure the GRFs with the skating technique. This system was able to measure three-dimensional (3D) GRFs, which means the force generated by medio-lateral movement was included as well, but skiers had to position the ski directly over the force plate. Moreover, only one ski contact for one trial could be recorded. Although the force measurement systems buried under snow did not influence the skiing technique, the movements were restricted to limited space. Therefore, more flexible ski force measurement equipment has emerged.

Several studies started to implement force transducers to the ski or roller ski bindings and measure the forces between ski boots and skis (or roller skis). Small force plates were implemented in the bindings introduced by Komi [1]. The vertical and anterior–posterior forces could be measured while skiing on snow, but they could not be used in the skate skiing technique which contains medio-lateral movements. Similarly, small force plates have also been implemented to roller ski bindings [5]. The vertical and medio-lateral forces were measured, but one equipped roller ski was about 50% heavier than a normal roller ski. In some studies, strain gauges have been installed on the bindings and measured the forces in several dimensions [6,7,8]. The force measurement bindings developed by Ohtonen et al. [8] have been used with both skis on snow [9,10,11,12] and roller skis on a treadmill [13]. The extra weight and height added by these bindings may, however, affect the skier’s performance on the treadmill using a roller ski [13]. The pressure insoles have also been used in several studies [14,15,16,17], but only vertical forces could be obtained.

For most skiers, roller skiing is one primary form of training method during the dry land training season [5] and is a ski-specific laboratory testing model that could reveal skiing technique in more detail [18]. Therefore, instrumented roller skis have also been investigated in previous studies [19,20]. The strain gauges were installed on the roller skis directly to measure the vertical [19,20] and horizontal [19] forces. However, there is also movement in the medio-lateral direction in skate skiing techniques. Thus, instrumented force measurement roller skis which could measure medio-lateral forces may be of good help for investigating the relevant skating techniques in cross-country skiing.

Consequently, the main aim of this present study was to validate a pair of newly designed two-dimensional (2D) force measurement roller skis. This pair of roller skis were first calibrated by the Technical Research Center of Finland (VTT MIKES, Kajaani, Finland). Then, forces measured by the roller skis were resolved in the global coordinate system (GCS) [20], and the accuracy of the force measurement roller ski would be checked by comparing forces measured by the roller skis and forces measured by a 3D force plate with a static and a simulated skating push-off test. To demonstrate whether the force measurement roller ski would affect roller skiing performance on a treadmill, a maximum speed test with the V2 technique would be performed by using both normal and force measurement roller skis.

## 2. Materials and Methods

### 2.1. Construction of the Force Measurement Roller Ski

**Figure 1:**
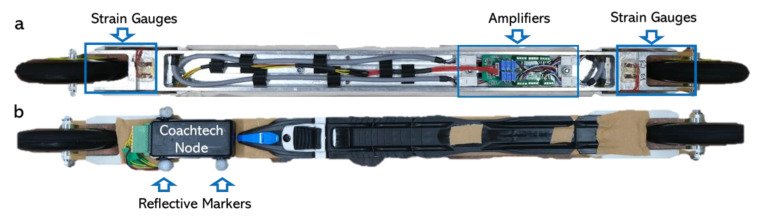
Construction of the force measurement roller ski. (**a**) Bottom view of the force measurement roller ski. (**b**) Top view of the force measurement roller ski.

**Figure 2:**
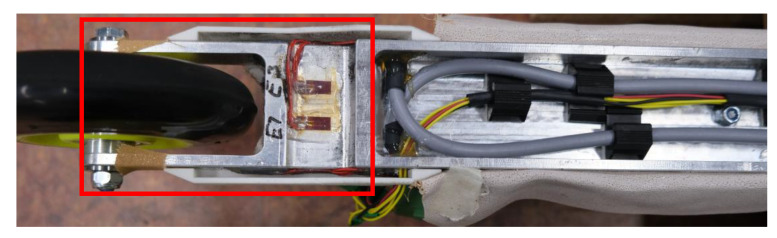
Diagram of strain gauge installation area.

The force measurement roller skis were designed and made by the Technical Research Centre of Finland (VTT MIKES, Kajaani, Finland). A custom-made aluminum alloy frame of the roller ski has been designed using the finite element method (FEM). Finite element analysis (FEA) has been used for both dimensioning the frame and determining the location of strain gauges on the roller ski body. Both roller skis contain four full-bridge strain gauge configurations (Figure 1 and Figure 2). There are four measurement channels for both roller skis. Two of these channels measure vertical forces (front and rear) and the other two measure medio-lateral forces (front and rear). The applied force causes a change in strain gauge resistance which causes a change in voltage, which can be measured from the Wheatstone full-bridge configuration. The amplifiers (Figure 1a) are embedded in the body of the roller skis and the voltage-level signals were acquired by the nodes (Figure 1b, Sports Technology Unit Vuokatti, University of Jyväskylä, Finland) of the Coachtech online measurement and feedback system [21], which were attached to the front part of the roller skis. The weight for one force measurement roller ski equipped with the Coachtech node was 1352 g.

### 2.2. Calibration and Force Calculation of the Force Measurement Roller Ski

The force measurement roller skis were calibrated in June 2022 before this validation measurement by the Technical Research Centre of Finland (VTT MIKES, Kajaani, Finland). The strain gauges were calibrated for a vertical force with forces from 0 N to 1000 N and for medio-lateral forces with forces from 0 N to 400 N. Three different types of tests (the load-up test, the signal-to-noise test, and the creep test) were performed for all force measurement roller ski sensors and for both vertical and medio-lateral directions. In the case of medio-lateral force, loading was performed from both sides. In the load-up test (the same for the vertical and medio-lateral directions), three preloads precede two increasing loads which are followed by increasing and decreasing loads. The measurement cycle between each load was 60 s. In the signal-to-noise test, voltage levels were measured without applied load. The measurement time was 180 s and the sampling frequency was 0.5 Hz. In the creep test, a 1000 N force was applied three times for both roller skis. Forces were applied directly from 0 N to 1000 N. The measurement cycle was 180 s. The creep was determined from the last load cycle. From the calibration process, the calibration factor and measurement uncertainty for the calibration process is calculated. The following uncertainty components are used in uncertainty calculations including calibration force, repeatability, resolution of the display device, creep of the instrument, zero-point fluctuation, hysteresis, interpolation error, and crosstalk. A linear model was used to calculate the calibration factor (N/mV) for each sensor. In addition to the linear model, second-order and third-order models had been tried as well, but the errors were not significantly reduced. The calibration factor (N/mV) used in this study for each strain gauge was shown in Table 1. The force from each signal channel ($F_{i}$) was calculated with the equation $F_{i}=a_{i}*U_{i}$, where $U_{i}$ is the voltage of signal channel i (mV) and $a_{i}$ is the calibration factor (N/mV). The total force of each direction (vertical or medio-lateral) could be derived with the equation $F_{sum}=F_{front}+F_{rear}$, where $F_{sum}$ represents the total force in one direction and $F_{front}$ and $F_{rear}$ are the forces in this direction from the front suspension and the rear suspension, respectively. In the measurements, only the sum of the forces was used.

### 2.3. Validation of the Force Measurement Roller Skis

#### 2.3.1. Static Test

**Figure 3:**
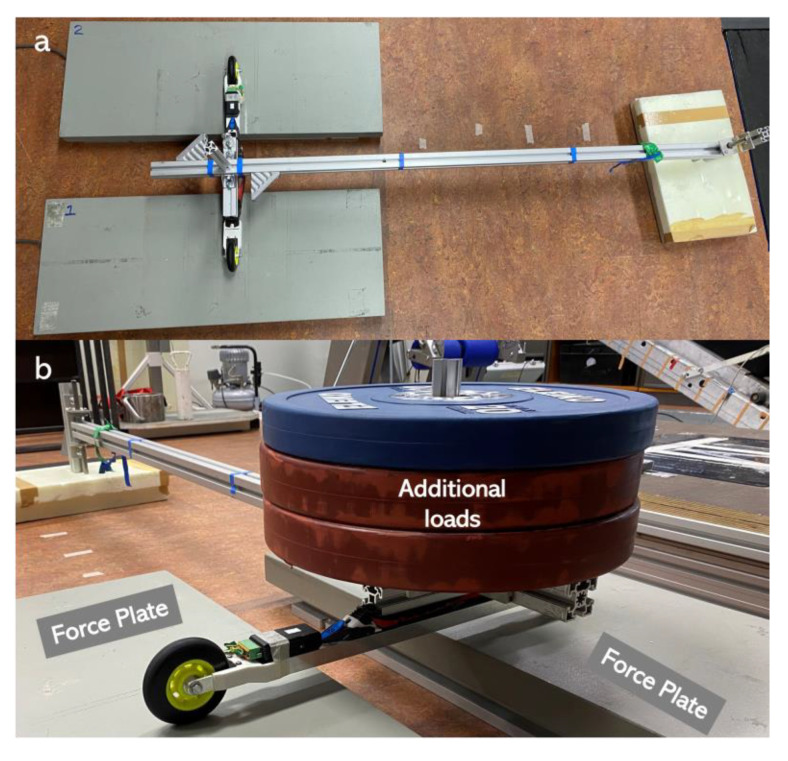
Diagram of the static test. (**a**) Custom-made equipment for placing the additional loads. (**b**) Static test with 70 kg additional loads.

The static tests were carried out on two AMTI 3D force plates (AMTI, Watertown, MA, USA) and were conducted for each roller ski (left and right) separately. The AMTI force plates were calibrated on 7 June 2022. Each time, one roller ski was placed with one wheel on each force plate by using custom-made equipment (Figure 3a). A total of 15 (0 kg to 150 kg) loads were placed with full weight on the roller ski. The force plates measured the forces in three directions and contained the forces induced by the weight of the roller ski and the custom-made equipment (Figure 3b). Therefore, the resultant force measured by the force measurement roller ski in this study should be equal to the resultant forces measured by the force plate minus the weight of the roller ski and the equipment. 

Forces measured by the roller ski were collected by the Coachtech online measurement and feedback system [21] at a sample rate of 400 Hz. Forces measured by the AMTI force plates were collected by the AMTINetForce Version 3.5.2 (AMTI, Watertown, MA, USA) with a sample rate of 1000 Hz. All force signals for each load were collected for at least 10 s. An average of 3 s of data was used to represent the forces under each load. The accuracy of the force measurement roller ski was quantified by using the relative difference in resultant forces between the force measurement roller ski and the AMTI force plates.

#### 2.3.2. Simulated Skating Push-Off Jump Test

**Figure 4:**
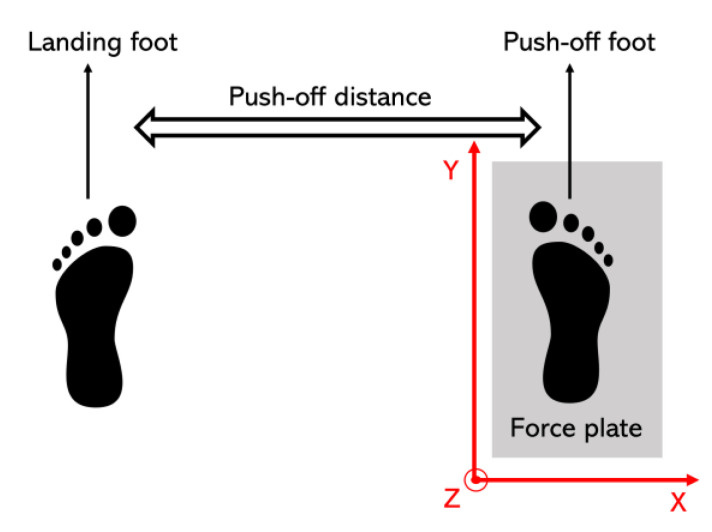
The definition of the push-off distance and the direction of the global coordinate system (GCS).

In order to test the force measurement roller ski in an applied dynamic situation, a simulated skating push-off jump test was performed over one AMTI 3D force plate (AMTI, Watertown, MA, USA). One male (age: 43 years; height: 183 cm; and weight: 83 kg) and one female (age: 27 years; height: 165 cm; and weight: 55 kg) highly skilled skier took part in this test. The maximum push-off jump distances were first found. The push-off distance was defined as the distance between the push-off foot and the landing foot (Figure 4). The maximum push-off distance for the male subject was 1.64 m with the right foot and 1.70 m with the left foot. The maximum push-off distance for the female subject was 1.49 m with the right foot and 1.45 m with the left foot. The push-off load was changed by changing the target push-off distance and the subject. The target distances were 65%, 75%, 85%, and 100% of the maximum push-off jump distance. Ten jumps at each target distance were recorded for further analysis. From a security perspective, subjects wore their normal training shoes with the landing foot. The force measurement roller ski was used by the push-off foot. The simulated skating push-off jump test was performed by both feet (left and right) separately.

Forces measured by the roller ski were collected by the Coachtech system [21] at a sample rate of 1000 Hz. Forces measured by the AMTI force plates were collected by the AMTINetForce Version 3.5.2 (AMTI, Watertown, MA, USA) with a sample rate of 1000 Hz. Three passive reflective markers were attached to the force measurement roller ski (Figure 1b) to record the position of the roller ski and were used to transform forces measured by the roller ski into the GCS [13]. The markers’ displacement was sampled at 250 Hz by using the Vicon motion capture system (Vicon, Oxford, UK). The weight for one force measurement roller ski with the node and the markers was 1358 g.

**Figure 5:**
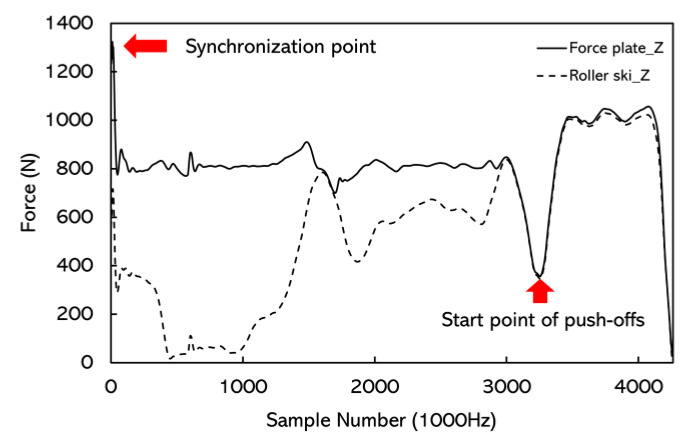
The synchronization of the signals and the start point of the simulated skating push-off. Force curves were from the male subject’s right foot push-off and the target push-off distance was the maximum push-off distance. The difference in the force curves before the start point was due to the position of the landing leg. The subject stood on the force plate with both legs before the push-off. The whole body weight was on the force plate but half or less body weight was on the roller ski; therefore, the signals do not match.

The force signal and the marker displacement signal were synchronized manually by using the rapid synchronization hit with the force measurement roller ski on the force plate before each push-off jump (Figure 5). The start of the push-off was defined as the vertical force minima during the unweighting phase. The end of the push-off was defined when the magnitude of the vertical force measured by the force plate was under 5 N. Forces measured directly by the force plate in the GCS (Figure 4) were treated as the reference value. As the movement in the Y direction during the simulated skating push-offs was small, forces from the force measurement roller ski and the force plate were not compared in the Y direction. The coefficient of multiple correlations (CMC, 0 < CMC < 1) [22,23,24] was calculated using MATLAB R2018a (MathWorks, Natick, MA, USA) from time-normalized curves between the force measurement roller ski and the force plate. The accuracy of the force measurement roller ski was quantified by using the absolute difference in forces between the force measurement roller ski and the force plates in the X and Z directions. 

#### 2.3.3. Practical Application

To demonstrate the practical application of the force measurement roller skis, one male (age: 24 years; height: 179 cm; and weight: 81.5 kg) and one female (age: 26 years; height: 166.5 cm; and weight: 55.5 kg) skier were roller skiing on a treadmill using the force measurement roller skis and using the reference roller skis (Marwe, SKATING 620 XC, wheel No. 0). The force measurement and the reference roller skis had the same wheels. The main aim of this test was to find whether the extra weight of the force measurement roller ski would affect skiing performance. 

For each subject, the following protocol was performed for two rounds and there was a 5 min rest in between. The incline of the treadmill for the female subject was 2° and for the male subject was 3°. The start speed of the treadmill was 18 km/h and increased by 1 km/h every 15 s. The treadmill stopped when the skier cannot keep up with the treadmill speed. The duration and the final speed were recorded as well as the cycle time, cycle rate, cycle length, and ski contact time were also obtained by using the accelerometer attached to the skis and poles with the Coachtech system [21]. The longer duration and greater final speed were used to present the performance. The protocol was performed twice within one week on different days by each subject, once with a pair of reference roller skis and another with the pair of force measurement roller skis.

The weight of the reference roller ski was 1025 g, which is 333 g lighter than the force measurement roller ski equipped with a node. As the Coachtech node is essential to the collection of the force signal, the balance point of a force measurement roller ski was measured with the Coachtech node attached to the front part of the roller ski. The balance point of the force measurement roller ski was moved 1.60 cm forward when compared to the reference roller ski. The torque around the ski boot attach point of the roller ski was calculated by using the gravitational force of the roller ski multiplied by the distance between the balance point and the ski boot attach point. The torque for the force measurement roller ski was 0.60 N·m and was 0.61 N·m for the reference roller ski. 

## 3. Results

**Figure 6:**
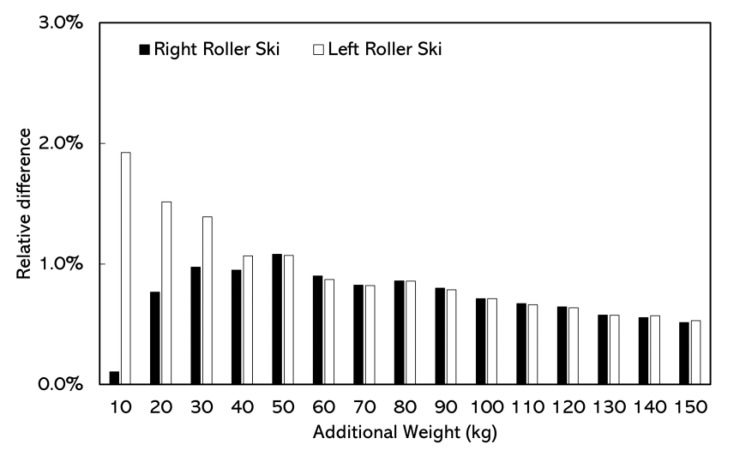
The absolute difference in resultant forces between force measurement roller ski and the AMTI force plates in static test at different additional loads.

In the static test, the relative difference in resultant forces between the force measurement roller ski and the AMTI force plate was lower than 2.0% (0.11~1.92%). The maximum relative difference in resultant forces was 1.92% when the additional weight was 10 kg (Figure 6).

**Figure 7:**
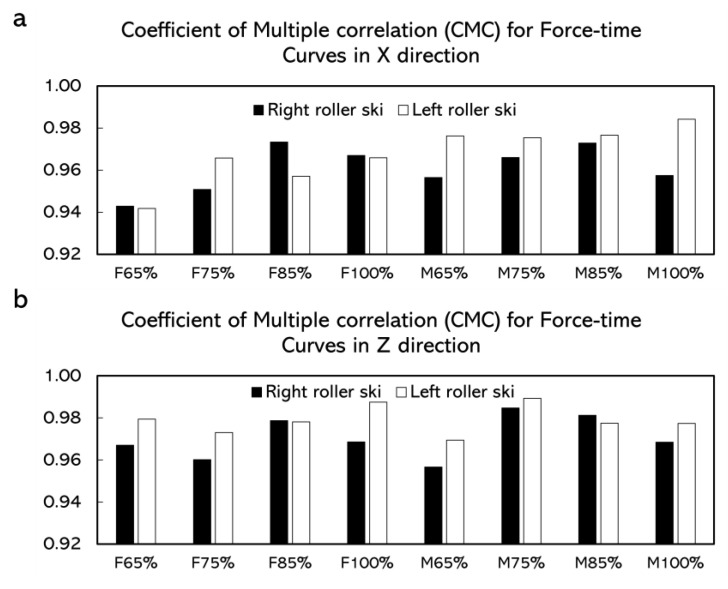
The coefficient of multiple correlations (CMC) for time normalized force-time curve. (**a**) CMC between force-time curves measured by force measurement roller ski and force plates in the X direction. (**b**) CMC between force-time curves measured by force measurement roller ski and force plates in the Z direction. F65%, F75%, F85%, and F100% represented female subjects and the target distance was 65%, 75%, 85%, and 100% of the maximum push-off distance, respectively. M65%, M75%, M85%, and M100% represented male subjects and the target distance was 65%, 75%, 85%, and 100% of the maximum push-off distance, respectively.

**Figure 8:**
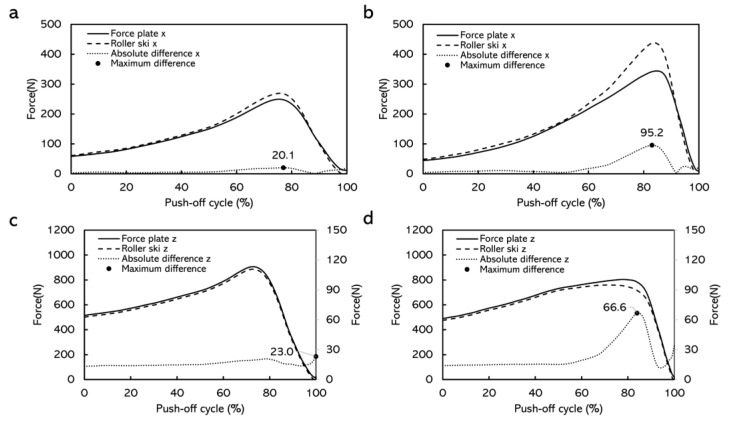
Comparison of force curves measured by force measurement roller ski versus force plate in the X direction and the Z direction with female subjects, and the absolute differences over time. (**a**) Force curves when the target distance was 65% of the maximum push-off distance. (**b**) Force curves when the target distance was 100% of the maximum push-off distance. (**c**) Force curves when the target distance was 65% of the maximum push-off distance. (**d**) Force curves when the target distance was 100% of the maximum push-off distance. Note: curves were averaged over 10 push-off cycles. The curves from these loads were chosen as examples having contained the highest and lowest maximum absolute differences.

**Figure 9:**
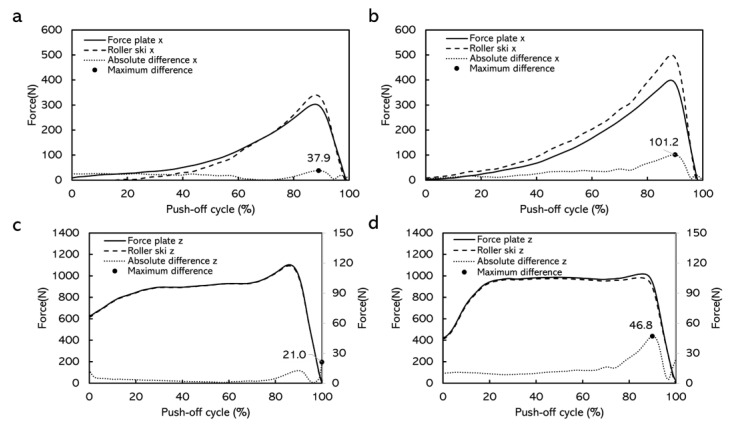
Comparison of force curves measured by force measurement roller ski versus force plate in the X direction and the Z direction with male subjects, and the absolute differences over time. (**a**) Force curves when the target distance was 65% of the maximum push-off distance. (**b**) Force curves when the target distance was 100% of the maximum push-off distance. (**c**) Force curves when the target distance was 65% of the maximum push-off distance. (**d**) Force curves when the target distance was 100% of the maximum push-off distance. Note: curves were averaged over 10 push-off cycles. The curves from these loads were chosen as examples having contained the highest and lowest maximum absolute differences.

In the simulated skating push-off test, the CMC values for force-time curves obtained from the force measurement roller ski and the force plate were generally above 0.940 (Figure 7). The average absolute differences for the forces in the X direction over one push-off cycle at different push-off loads were 8.5–33.3 N (Table 2). The average absolute differences for the forces in the Z direction at different push-off loads were 3.9–23.4 N (Table 3). The maximum absolute difference was 20.1–101.2 N in the X direction and was 21.0–66.6 N in the Z direction (Figure 8 and Figure 9).

**Figure 10:**
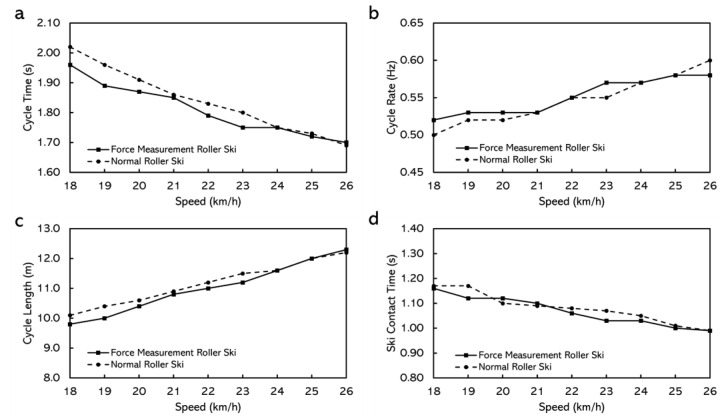
The cycle characteristics while using normal roller skis and force measurement roller skis for male subjects. (**a**) Cycle time. (**b**) Cycle rate. (**c**) Cycle length. (**d**) Ski contact time (from right roller ski).

**Figure 11:**
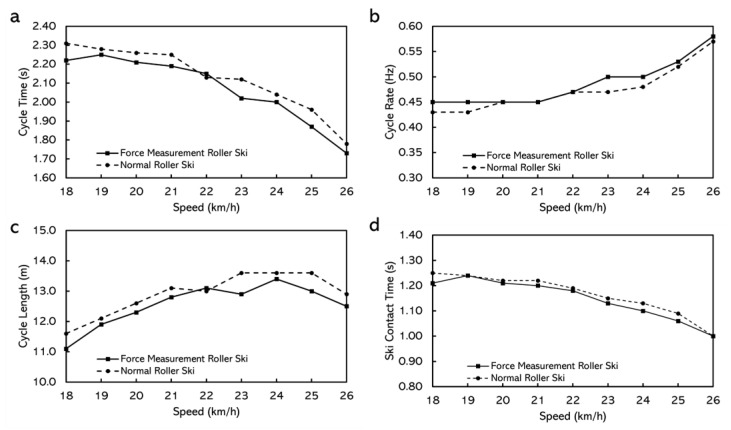
The cycle characteristics while using normal roller skis and force measurement roller skis for female subjects. (**a**) Cycle time. (**b**) Cycle rate. (**c**) Cycle length. (**d**) Ski contact time (from right roller ski).

When skiing on the treadmill, the durations for the tests did not have any major differences with different roller skis. Male skiers even had longer duration and better performance by using the force measurement roller ski (Table 4). The cycle characteristics, while using both roller skis at different speeds, are shown in Figure 10 and Figure 11. For the female skier, lower cycle rate, longer cycle length, and longer ski contact time were discovered by using the normal roller ski but for the male skier, the effects of the roller ski on cycle characteristics were not obvious (Figure 11).

## 4. Discussion

The force measurement roller ski used in this study was not the first one used in scientific studies. However, compared with the force measurement roller ski introduced in a previous study [20], these new roller skis can measure both vertical and medio-lateral forces which are more appropriate for the relevant skating techniques in cross-country skiing. The idea of this force measurement roller ski was from the force measurement bindings developed by Ohtonen et al. [8]. The binding was used in roller skiing on the treadmill [13] and the weight of one equipped roller ski was 1650 g, which is 27.6% heavier than the force measurement roller ski used in this current study. The Coachtech nodes placed between the binding and the front wheel of both roller skis were used for power supply and data transmission. This means that the data measured by the force measurement roller ski could be transported wirelessly via the Coachtech system. Therefore, from a construction point of view, this force measurement roller ski has the benefit of being lightweight and can wirelessly measure forces in more dimensions without any interfering cables and transmitters need to carry but subject. In addition, no extra height was added to the roller ski in the current study, which was reported as a problem in earlier studies [8]. The calibration factors used in this study were obtained in the calibration test carried out in June 2022. Another calibration test was in December 2020, and the calibration factors from this previous test can be found in the appendix (Appendix A). During these 18 months, the force measurement roller skis were used intensively by skiers to check the signal collection via the Coachtech system. The calibration factors used in this study did not change obviously when compared to the factors from the earlier calibration test, which indicated that the measurements could remain reliable and stable over several months. However, periodic calibration is recommended.

The static test was conducted to quantify the accuracy of the resultant forces obtained by the force measurement roller ski. The forces measured by the force plate also contained the weight of the roller ski and the custom-made frame; however, these weights were subtracted while doing the comparison. Although differences in relative resultant force difference were found between left and right force measurement roller skis at lower additional weights (10–30 kg), we are not focusing on the accuracy difference between left and right force measurement roller skis. In addition, the static test results are within measurement uncertainty for the vertical direction defined in the calibration process (Appendix B). The relative difference in resultant forces ranged from 0.11%~1.92% in this study. In a previous study, the difference of vertical resultant forces measured by the force plate and the instrumented one-dimensional roller ski ranged from 5.40% to 10.59% [20], which is greater than what we found in the present study. Possible reasons for improved accuracy may be due to the different construction of the force measurement roller skis.

The simulated skating push-off jump test was conducted to validate the force measurement roller ski in an applied dynamic situation. The CMC depicting the similarity between waveforms and the value of the CMC close to one implies that the curves involved were similar [13,22,23,24]. The CMC values in this study were generally above 0.940, which indicated that at each push-off load, the force-time curves obtained by the force plate and the force measurement roller ski after being transformed into the GCS were similar in each direction. Similar to the static test, the forces measured by the force plate contained the weight of the roller ski. However, the weight of the roller ski could not be subtracted during the dynamic test when comparing the force component in the GCS. Therefore, there must be some difference between the forces measured by the force plate and the forces measured by the roller ski. The average absolute difference for the forces in the Z direction at different push-off loads was 3.9–23.4 N (Table 3) and the maximum absolute difference was 21.0–66.6 N in the Z direction (Figure 8 and Figure 9). The result from a previous study shows that the leg vertical force change among one skate skiing cycle from sub-maximum speed up to maximum speed was about 60–1415 N [11]. Since the differences between the forces measured by the force measurement roller ski and the reference force plate in the present study are smaller than observed during different-intensity skiing, the accuracy of the forces measured by the force measurement roller ski can be considered to be high enough to be used in practice e.g., for skiing technique observations. Although it is impossible to have the forces measured by the force measurement roller ski in full accord with forces measured by the force plate, the differences can be considered promising and acceptable. Figure 8 and Figure 9 presented the absolute differences over time. The absolute differences were constant before the maximum push-off forces appeared. Moreover, the maximum absolute differences generally appeared around the maximum push-off force or at the end of the push-off. This may be due to the inconsistency of the force change from these two different force measurement systems. In cross-country skiing, the heel of the ski boot is not fixed on the roller ski. When the heel of the ski boot is about to go off the roller ski, the resistance of the strain gauge on the force measurement roller ski may change, thereafter leading to the change in forces. Since the full weight of the subject and the roller ski were still on the force plate, the forces measured by the force plate may not change. This inconsistency may lead to a change in the absolute difference over time. In addition, force transmission parts typically in calibrations are made from steel but, in this case, the force transmission parts are the rubber wheels which may also affect the difference in forces measured by the force measurement roller ski and the force plate. The absolute differences between these two force measurement systems in this dynamic test were greater than that in the static test. This is possibly caused by the direction of the applied force. The force measurement roller ski measured the forces between the foot and the roller ski, and the force plate measured the forces between the roller ski and the force plate. When the subject was performing the push-off jump, the roller ski was edged. The applied force on the roller ski and the force plate may not be parallel to each other. The crosstalk from the vertical force channel into the medio-lateral channel may also be an effect that may influence the amplitude of the measured force in the medio-lateral channel. These may cause some errors when comparing the force component converted to the GCS. 

The extra weight of the force measurement roller ski did not affect the performance of the skiers. The duration skiers stayed on the treadmill and the final speed skiers could reach were not affected much by the roller skis they use. Although there was a 333 g difference between the roller skis, the balance point of the roller ski changed as well. This led to the torque difference around the ski boot attach point on the roller ski being 0.01 N·m, which could be considered negligible. Therefore, the extra weight of the force measurement roller ski appears to be acceptable to the athletes. However, the extra weight may still affect the cycle characteristics while roller skiing, especially for female athletes. This may be due to the lighter body weight and relatively lower muscle strength when comparing the female athletes with the male athletes. The male athlete even seem to have a better performance by using the force measurement roller ski. This may be because the stiffness of the force measurement roller ski suited her better. The body of the force measurement roller ski is made of aluminum and the body of the reference roller ski is a honeycomb wooden structure. The stiffness of the two bodies may have some difference and, thereafter, affect the performance. 

## 5. Conclusions

This developed instrumentation where the resistance strain gauges were mounted to the suspensions of the roller ski wheels is a practicable tool for measuring the magnitude of the forces applied on the roller skis in two dimensions in skate skiing. Markers attached to the roller skis can help transform the measured forces into the global coordinate system. Even though the transformed force component measured by the force measurement roller ski did not fully match the forces measured by the reference force plate, the possible reasons for the differences were analyzed. Despite small differences between the measurement systems, the derived forces in the X and Z directions can be considered valid and reliable. The extra weight of the force measurement roller ski has a small effect on the skier’s roller skiing performance. Therefore, this instrumented force measurement roller ski can be useful for future research during daily training. One limitation of this validation study was that the validity of the force in the Y direction was not examined. In addition, skiers who participated in the practical application test were all adult skiers; whether the force measurement roller ski would have effects on roller skiing performance for junior and adolescent skiers needs further investigation.

## Figures and Tables

**Table 1:** Calibration factors (N/mV) for each strain gauge mounted on the roller skis.

Roller Ski	Strain Gauge	Calibration Factor
Right roller ski		
	Front vertical	0.2444
	Front medio-lateral	0.1170
	Rear vertical	0.2418
	Rear medio-lateral	0.1154
Left roller ski		
	Front vertical	0.2452
	Front medio-lateral	0.1178
	Rear vertical	0.2489
	Rear medio-lateral	0.1157

**Table 2:** The absolute difference (N) between force-time curves obtained by the AMTI force plate and the force measurement roller ski system in the X direction.

Loads	Right Roller Ski	Left Roller Ski
F65%	8.5 ± 5.6	18.8 ± 14.2
F75%	15.4 ± 12.3	26.1 ± 20.9
F75%	22.1 ± 17.0	28.2 ± 21.3
F100%	23.4 ± 26.5	32.5 ± 25.4
M65%	22.2 ± 10.2	18.8 ± 9.2
M75%	24.4 ± 12.6	19.4 ± 9.1
M85%	30.6 ± 19.0	21.1 ± 15.9
M100%	33.3 ± 25.0	24.4 ± 17.4

Note: The absolute differences were averaged over 10 push-off cycles. F65%, F75%, F85%, and F100% represented female subjects and the target distance was 65%, 75%, 85%, and 100% of the maximum push-off distance, respectively. M65%, M75%, M85%, and M100% represented male subjects and the target distance was 65%, 75%, 85%, and 100% of the maximum push-off distance, respectively.

**Table 3:** The absolute difference (N) between force-time curves obtained by the AMTI force plate and the force measurement roller ski system in the Z direction.

Loads	Right Roller Ski	Left Roller Ski
F65%	15.8 ± 2.2	9.9 ± 3.3
F75%	18.3 ± 5.0	13.5 ± 7.1
F75%	20.7 ± 8.1	14.7 ± 8.5
F100%	23.4 ± 14.9	18.8 ± 13.4
M65%	11.7 ± 5.1	3.9 ± 3.4
M75%	11.7 ± 3.2	4.1 ± 4.0
M85%	13.8 ± 6.6	5.7 ± 7.2
M100%	15.2 ± 9.7	7.3 ± 9.8

Note: The absolute differences were averaged over 10 push-off cycles. F65%, F75%, F85%, and F100% represented female subjects and the target distance was 65%, 75%, 85%, and 100% of the maximum push-off distance, respectively. M65%, M75%, M85%, and M100% represented male subjects and the target distance was 65%, 75%, 85%, and 100% of the maximum push-off distance, respectively.

**Table 4:** The duration (s) and the final speed by using the force measurement roller ski (FMR) and the normal roller ski (NR).

	Male		Female	
	FMR	NR	FMR	NR
Duration (s)	143	134	147	150
Final speed (km/h)	27	26	27	27

## Data Availability

Pseudonymized datasets are available to external collaborators subject to agreement on the terms of data use and publication of results. To request the data, please contact Vesa Linnamo (vesa.linnamo@jyu.fi).

## Appendix A. Calibration Factors for Each Strain Gauges from the Calibration Test in December 2020

**Table A1:** Calibration factors (N/mV) for each strain gauges mounted on the roller skis (from the calibration test in December 2020.

Roller Ski	Strain Gauge	Calibration Factor
Right roller ski		
	Front vertical	0.2428
	Front medio-lateral	0.1175
	Rear vertical	0.2431
	Rear medio-lateral	0.1148
Left roller ski		
	Front vertical	0.2442
	Front medio-lateral	0.1174
	Rear vertical	0.2459
	Rear medio-lateral	0.1161

## Appendix B. Vertical Force Combined Expanded Relative Measurement Uncertainty for Different Loading

**Table A2:** Vertical force combined expanded relative measurement uncertainty (%) for different loading.

	Right Roller Ski	Left Roller Ski
Force (kN)	W (k = 2)	W (k = 2)
0.2	3.5	4.5
0.4	3.4	3.6
0.6	2.7	2.9
0.7	2.5	2.7
0.8	2.3	2.2
0.9	1.0	1.4
1.0	0.4	0.8
